# Health Belief Model (HBM) and vaccination during pandemics

**DOI:** 10.1192/j.eurpsy.2022.786

**Published:** 2022-09-01

**Authors:** A. Zartaloudi

**Affiliations:** University of West Attica, Nursing, Athens, Greece

**Keywords:** Covid-19 pandemic, Health Belief Model, vaccination behavior, vaccination intentions

## Abstract

**Introduction:**

With the COVID-19 pandemic recognized as a major threat to human health, promoting vaccination is of paramount importance to public health.

**Objectives:**

To examine the association between factors of the Health Belief Model (HBM) and intentions to be vaccinated against COVID-19, when a vaccine becomes available.

**Methods:**

A literature review has been made through PubMed database.

**Results:**

The HBM dimensions “perceived barriers”, “perceived benefits” and “perceived severity” were considered to be significant predictors of acceptance of vaccinations. The HBM constructs of cues to action (trust in third-party information sources), perceived severity of and susceptibility to COVID-19, and beliefs about the protection benefits of a COVID-19 vaccine, subsequently may elicit willingness to vaccinate. Individual predictors of vaccination were believing the vaccine is effective at preventing COVID-19, recalling their doctor recommending the vaccine. Common perceived barriers against vaccination included believing the vaccine could give people the virus, believing the vaccine can make individuals ill afterwards and preferring to develop immunity “naturally”. Patients who delayed and refused vaccine doses were more likely to have vaccine safety concerns and perceive fewer benefits associated with vaccines.

**Conclusions:**

HBM is an effective tool for identifying facilitators and barriers to health behaviors. Health promotion should make use of the HBM, as the model provides a theoretically understanding of the dynamics that may enable the success of important health-related policy in the wake of COVID-19 and future pandemics and identifies the communication mechanisms that must be leveraged by governments and authorities in enforcing policy.

**Disclosure:**

No significant relationships.

